# Evidence of dengue virus replication in a non-traumatic spleen rupture case

**DOI:** 10.1007/s00705-017-3527-6

**Published:** 2017-08-14

**Authors:** Luiz José de Souza, João de Azevedo, Liza Ingride Acha Kohler, Lorena de Freitas Barros, Mariana Arêdes Lima, Emiliana Mandarano Silva, Ronaldo Mohana-Borges, Priscila Conrado Guerra Nunes, Marciano Viana Paes

**Affiliations:** 1 Centro de Referência de Doenças Imunoinfecciosas, Campos dos Goytacazes, RJ Brazil; 2 Hospital dos Plantadores de Cana, Campos dos Goytacazes, RJ Brazil; 3 Faculdade de Medicina de Campos, Campos dos Goytacazes, RJ Brazil; 4 Laboratório de Genômica Estrutural, Instituto de Biofísica Carlos Chagas Filho, Universidade Federal do Rio de Janeiro, Rio de Janeiro, RJ Brazil; 5 Laboratório de Imunologia Viral, Instituto Oswaldo Cruz, Fundação Oswaldo Cruz, Rio de Janeiro, Brazil; 6 Laboratório Interdisciplinar de Pesquisa Médica, Instituto Oswaldo Cruz, Fundação Oswaldo Cruz, Rio de Janeiro, Brazil

**Keywords:** Splenic rupture, Dengue, NS3 detection, Acute abdomen

## Abstract

The present report describes a case of splenic rupture due to dengue, a rare complication of dengue that should be considered in any patient with suspected dengue disease who started with left upper quadrant abdominal pain and hypotension. The pathophysiology of this entity is not yet well elucidated, but one of the theories present in the literature is that it is due to a depletion of coagulation factors and platelets leading to intra-splenic hemorrhage and rupture. The RT-PCR technique detected serotype 1 and histopathological studies of the spleen revealed significant atrophy of lymphoid follicles and extensive hemorrhage areas. Besides histopathological observations, virus replication was investigated by detection of dengue antigens, especially the non-structural 3 protein (NS3) in endothelial cells and splenic macrophages. This important complication has serious clinical repercussions and high mortality, due to the diagnostic difficulty and many factors that usually confuse or delay its diagnosis. Therefore, it is of the utmost importance to recognize their manifestations and their management to try to best minimize their consequences and mortality.

## Introduction

Dengue virus (DENV) is an arbovirus, classifiable in the *Flaviviridae* family, which has four distinct serotypes (DENV 1–4) [1]. The viral RNA genome encodes three structural proteins: capsid, membrane and envelope, and seven non-structural proteins: NS1, NS2A, NS2B, NS3, NS4A, NS4B, and NS5 [2]. The disease caused by DENV is classified as classical dengue and dengue hemorrhagic fever; however, a new classification was recently recommended: dengue fever without signs of alarm, dengue fever with signs of severe dengue fever [3].

Dengue is an infection that can lead to several complications, such as neurological (encephalitis, stroke, Guillain-Barre syndrome), cardiac (myocarditis), gastrointestinal (liver failure, acute acalculous cholecystitis) or even an acute abdomen [4]. Spontaneous rupture is a very rare condition independent of etiology, even rarer when resulting from dengue infection. It is most common in infections such as malaria, typhoid fever and infectious mononucleosis. The mechanism underpinning acute abdomen formation is still unclear, but it is believed to be due to depletion of coagulation factors and platelets leading to intra-splenic hemorrhage and its consequent rupture [5].

This is a descriptive study, in the form of a case report, of a spontaneous splenic rupture in a patient hospitalized with a dengue infection at Hospital Plantadores de Cana, Campos dos Goytacazes in Rio de Janeiro. The objective is to discuss the clinical and virology aspects of splenic rupture that relate to dengue virus disease.

## Case report

In August 2015, a 23-year-old male patient developed anorexia, headache, retro-orbital pain, fever, myalgia, prostration/ asthenia, and dry cough for four days. He presented at the Plantadores de Cana Hospital because of diffuse abdominal pain. The patient denied having comorbidities, previous surgeries, and use of medications, alcohol, tobacco, illicit drugs, athletic activity, intense physical effort or history of trauma. He was admitted for exams and clinical support. Upon physical examination, the abdomen was painful to diffuse palpation examination, with no other changes in other systems. Laboratory tests revealed only mild thrombocytopenia (119,000/mm^3^). After the third day of hospitalization, there was a worsening of the condition, with left upper quadrant abdominal pain, tachypnea, tachycardia, jaundice, dehydration, signs of peritoneal irritation and shock. Laboratory investigations showed the following: leukocytosis (15,490/mm^3^), 34% of hematocrit, hemoglobin dosage of 12,5 g/dL, intense thrombocytopenia (29,000/mm^3^), blood urea was 24 mg/dl, serum creatinine of 0,9 mg/dl, aspartate transaminase (AST) of 591 U/L, alanine transaminase (ALT) of 450 U/L, total bilirubin of 3,1 mg/dl (direct bilirubin- 1,9 mg/dl), 90 U/L amylase and 67 U/L lipase. The patient was maintained with clinical support, awaiting elevation of platelet dosage for laparotomy. On the sixth day of hospitalization, the blood count for leukocytosis was the same (15,490/mm^3^) and there was a partial improvement of thrombocytopenia (102,000/mm^3^). We performed abdominal computed tomography scan (CT scan), serology and RT-PCR for dengue. Serology for infectious mononucleosis and other infectious differential diagnoses were not performed. The patient was referred for a laparotomy, and the removed spleen was submitted to histopathological examination and immunohistochemistry for dengue. After the splenectomy, the patient received all the recommended treatment and was clinically well following outpatient follow-up.

## Material and methods

### Ethical procedures

All procedures performed during this work were approved by the Ethics Committee of the Oswaldo Cruz Foundation/FIOCRUZ, with the number CAEE: 5722141010015248 for studies with dengue fatal cases and controls. This consent procedure was approved by the ethics committee. The consent for the publication of this case report and any additional related information was taken from the patient involved in the study.

### CT scan

The abdominal analysis was performed by using a computed tomography scanner HiSpeed machine by General Electric Co.

### Dengue diagnosis

For dengue diagnosis, IgM antibody capture ELISA (MAC-ELISA) was performed by using the Panbio dengue IgM Capture ELISA (Panbio Diagnostics, Queensland, Australia). Viral RNA was extracted from sera using the QIAamp Viral RNA Mini kit (Qiagen, Valencia, CA) and RT-PCR for detecting and typing DENV was performed as described previously Lanciotti [1].

### Histopathological analysis and immunohistochemistry of the spleen tissue

A spleen sample from the patient biopsy was paraffin-embedded, fixed in 10% formalin, cut (4 µm), deparaffinized in xylene and rehydrated with alcohol, as described elsewhere by Póvoa [6]. The sections were analyzed by staining with hematoxylin and eosin (HE) and visualized using a Nikon ECLIPSE E600 microscope. Immunohistochemistry assays for detection of NS3 antigens were performed according to the protocol described by Póvoa [6]. The anti-DENV-3 polyclonal antibody (raised in Swiss mouse inoculated with DENV-3) was used for NS3 detection.

The negative control spleen was obtained from a patient whose cause of death was non-dengue related (or other infectious diseases), and was provided by Dr Carlos Basilio de Oliveira of the Pathological Anatomy, Gaffrée Guinle Hospital, University of Rio de Janeiro.

## Results

**Fig. 1:**
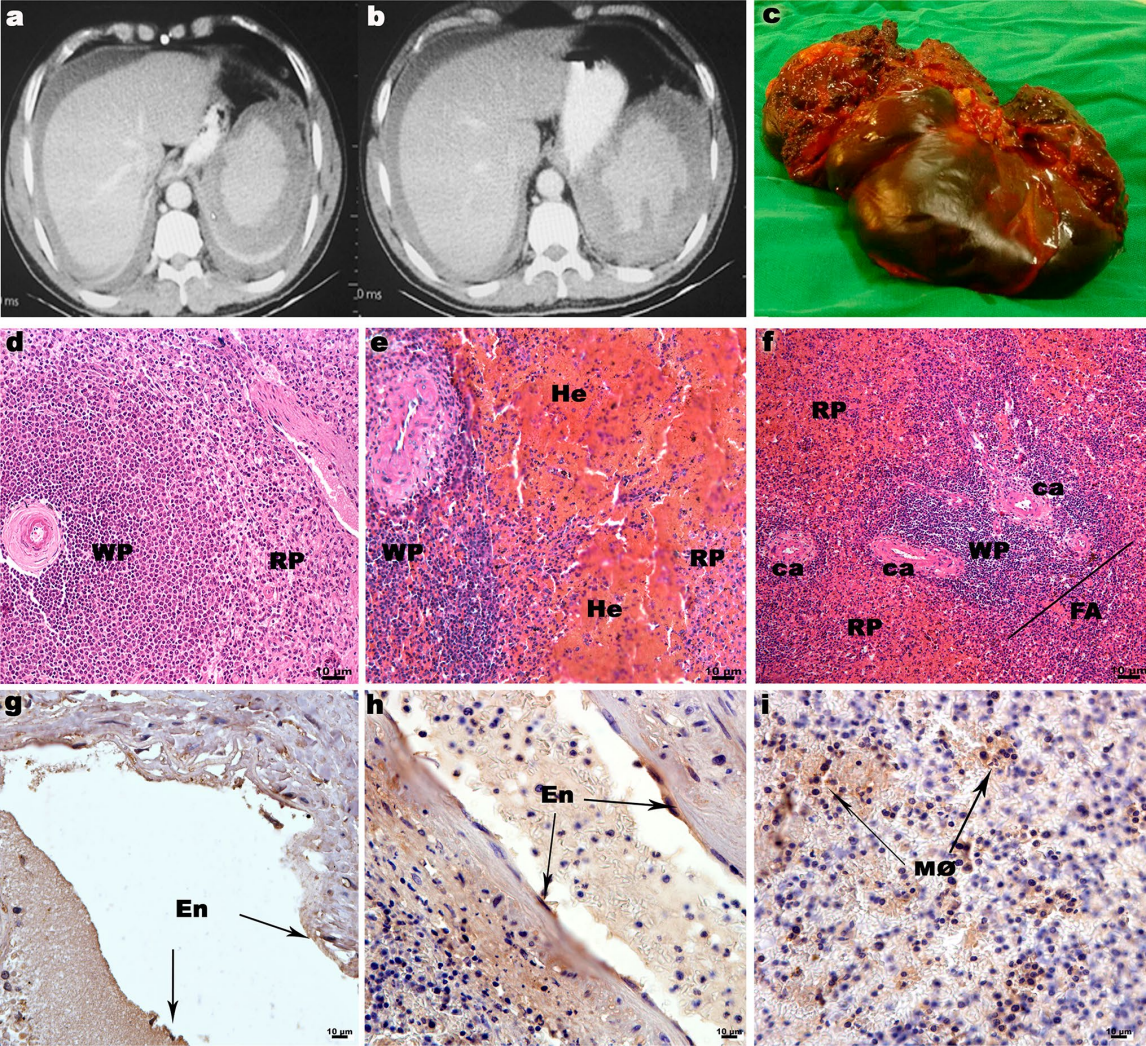
Tomographic and anatomopathological image of the spleen in this dengue case, exhibiting splenic alterations. A and B: Computed tomography showing splenic rupture with sub-capsular hematoma and hemoperitoneum. C: Ruptured spleen removed by laparotomy. D-I Histopathological analysis of the spleen tissue: (D): Negative control spleen stained with Hematoxylin and Eosin showing regular areas of white pulp (WP) and red pulp (RP). (E and F): The Dengue case spleen showing large areas of hemorrhage (He) in the red pulp region (Rd) and demonstrable follicular atrophy (FA). (G): A spleen biopsy from the negative control stained with NS3 monoclonal antibody. Non-stained endothelium cells (En). (H-I): Positive identification of DENV antigen in the DENV-infected spleen in En and circulating macrophages (Mø). Central artery (CA)

Computed tomography of the abdomen showed an enlarged spleen, which was dysmorphic, with a markedly irregular contour, with circumferential sub-capsular collection, a compatible aspect with restrained splenic rupture and formation of subcapsular hematoma, as well as moderate amounts of free liquid in the peritoneal cavity with high density, compatible with hemoperitoneum (figure 1A and B).

IgM serology for dengue on the ninth day of disease progression was positive and RT-PCR demonstrated dengue virus serotype 1.

The spleen that was removed (Figure 1C) showed congestion of the red pulp and an area of subcapsular coagulant necrosis, acute splenitis and a capsule with deposition of fibrin.

The histological section of the spleen obtained from this case of dengue showed extensive areas of hemorrhage in the red pulp region and demonstrable follicular atrophy in the central arteriole (Figure 1E and 1F), in contrast to the negative control (Figure 1D). The immunohistochemistry analysis, using antibodies against the NS3 non-structural protein, detected virus antigens in endothelial cells and splenic macrophages (Figure 1H and 1I).

## Discussion

Dengue is currently one of the most important diseases in the world. The epidemiology of dengue has been characterized by an increase in incidence and spread, resulting in a large number of severe cases [7]. The viremic stage of dengue lasts from two to seven days and is characterized by a sudden onset of high fever associated with nonspecific symptoms such as headache, rash, prostration, myalgia, nausea, vomiting, retro-orbital pain, anorexia and generalized arthralgia. Between the third and seventh days of disease onset, when fever occurs, alarm signs usually arise: uncontrollable vomiting, intense and continuous abdominal pain, painful hepatomegaly, respiratory distress, excessive drowsiness or irritability, hypothermia, mucous membranes, decreased sweating, and cavitary effusions. The severe form of the disease can manifest itself with signs of organ dysfunction, shock and severe bleeding [4, 8].

The spleen is often congested in severe dengue, and sub-capsular hematomas are found in 15% of necropsies. However, splenic rupture in dengue infection is extremely rare and only a few cases have been reported in the literature [9].

Splenic rupture is a condition that can be post-traumatic in origin, which is more common, or non-traumatic, which is far rarer. The non-traumatic form can be pathological or spontaneous. The pathological term is applied to splenic disease that presents with abnormal histology, whereas spontaneous rupture occurs without histological changes [10]. Non-traumatic splenic rupture may result from infection, malignancy or connective tissue disease. Associated infections include mononucleosis, malaria, typhoid, endocarditis, aspergillosis, and dengue. Hematologic malignancies, such as myeloid leukemia, non-hodgkin’s lymphoma and chronic lymphocytic leukemia, as well as metastases of various tumors, such as choriocarcinoma, malignant melanoma, and teratoma, are prominent among the causes of malignancy. In the group of collagenoses, rheumatoid arthritis, SLE, polyarteritis nodosa and Wegener’s granulomatosis are also associated with splenic rupture [11, 12].

The pathophysiological mechanism of splenic rupture due to dengue is not yet well elucidated. The most accepted hypothesis suggests that the mechanism is subcapsular hemorrhage due to a combination of vascular abnormalities, decreased coagulation factors and severe thrombocytopenia [11, 13]. In the vast majority of cases, splenic rupture occurs in the viremic phase of dengue, that is, before the development of antibodies and in the presence of antigen. However, there is a report in the literature of splenic rupture due to dengue during the recovery phase of the disease, around the eighth day [14].

Abdominal pain is the most frequent symptom of splenic rupture. This is characterized by diffuse pain to being with followed by pain in the upper left abdomen, evolving to peritoneal irritation associated with hemorrhagic shock signs (hypotension, tachycardia and hematocrit value decrease). It is known that acute acalculous cholecystitis is a common and well-known complication of dengue, being a differential diagnosis for cases of splenic rupture. Acute acalculous cholecystitis also presents with abdominal pain, but this is associated with the upper right abdomen and a positive Murphy signal, with or without shock signs (extravasations shock, not hemorrhagic). Its treatment is conservative, unlike the great majority of cases of splenic rupture, in which the treatment is surgical, and of urgency. Thus, patients with dengue and abdominal pain should be better investigated. Hypotension, tachycardia and oliguria are clinical signs common in severe dengue, which happen due to the extravasations, but can also be indicative of bleeding from a possible splenic rupture in these patients [10, 11]. Splenic rupture associated shock can be easily misdiagnosed and should be distinguished from classic dengue shock syndrome. Both shocks develop hypotension and tachycardia. Splenic rupture shock, a hemorrhagic shock, presents hematocrit value decreases, while dengue shock syndrome, an extravasations shock, presents as increases in the hematocrits values [13].

The diagnosis of spleen rupture is confirmed by means of ultrasonography, nuclear magnetic resonance, computed tomography or peritoneal lavage diagnosis. Some authors consider paracentesis as the most effective way to perform the diagnosis, being useful for the detection of spontaneous splenic rupture and also in patients with suspected intraperitoneal and unstable hemorrhage [10]. Ultrasonography is an inexpensive and practical way to obtain a rapid diagnosis of intraperitoneal fluid or blood and can be performed in the bed or in the emergency unit. Computed tomography not only detects rupture, but also shows the degree of severity of the splenic lesion and the presence of free fluid in the abdominal cavity. Ultrasonography is an effective and non-invasive method with no risk for hemodynamically unstable patients, whereas tomography may be useful in hemodynamically stable patients [10, 15, 16]. We consider CT the most appropriate diagnosis test, but it depends on patient hemodynamic stability and its accessibility, which may not be the case in risk areas for dengue fever, which are usually low income areas.

Histopathology demonstrated the characteristic hemorrhagic process of spleen rupture, but we also observed the atrophy of lymphoid follicles (rich region of T and B cells) that has already been described in the literature as a characteristic of dengue infection [6]. During the DENV replication process, non-structural proteins participate in a regulatory and essential way for viral perpetuation. NS3 proteins plays a role as the viral protease, where it cleaves the polyprotein into structural and non-structural proteins, making it essential for viral survival [2, 17, 18]. This same protein was identified as a target for antivirals against flaviviruses [19]. The detection of DENV NS3 protein in macrophages and endothelial cells leads to the hypothesis that viral replication occurs in these cells, since the dengue virus has a tropism for this organ [20].

Management of patients with spontaneous splenic rupture is well debated. Of the 136 cases of pathological splenic rupture reported in the literature, 88 received surgical intervention. Of these, 55 (63%) survived and 33 (37%) died. Of the 43 patients who had a conservative approach, 40 died [21]. It is notable that surgical treatment not only controls bleeding but also contributes to the almost immediate resolution of thrombocytopenia, reestablishing hemostasis in about two days. The current recommendation in most cases of dengue infection is still splenic surgical removal (splenectomy). However, other recent studies have demonstrated favorable results with conservative treatment in non-severe patients [10, 13]. Therefore, the correct therapeutic choice depends on the hemodynamic status of the patient.
